# Supplementary material for: The EmojiGrid as a rating tool for the affective appraisal of touch
Source: PLoS One. 2020 Sep 2;15(9):e0237873. doi: 10.1371/journal.pone.0237873 (PMC7467219; doi:10.1371/journal.pone.0237873)
Supplement: Attachment — Submitted filename: Reply_to_reviewers.docx [file pone.0237873.s002.docx]

| Comments of Reviewer 1 | Authors reply |
| --- | --- |
| **Abstract** (lines 19-21) “We conclude that the EmojiGrid can also serve as a valid and efficient graphical self-report instrument to measure human affective response to a wide range of (possibly mediated) tactile signals.” In this context, without reference to any relevant background literature, it isn’t clear what “mediated” tactile signals are. The authors could consider removing the term from the abstract or revising to make its meaning clearer. When the same term is used in the introduction it is a little more clear because of the context the term is being used in. | We agree with the reviewer that the term “mediated” is not clear without further explanation, and therefore removed it from the abstract. |
| **Introduction** (lines 24 – 40)- In this first paragraph of the introduction the authors should more explicitly define what they mean by affective touch. Initially it seems to refer to an emotional evaluation of a tactile stimulus – eg the properties of a fabric. However, it is then related to the specific response properties of a class of cutaneous afferent found in the hairy skin of mammals which responds optimally to low force and velocity touch. Affective touch does not necessarily have to mean C-tactile afferent activating touch and the authors are best to define it with relevance to their own study. For example, exploration and evaluation of a soft material with the fingertips and palms of the hands might be termed affective touch but it doesn’t involve activation of C-tactile afferents. | We added the definition provided by Morrison (2016):  “ Affective touch has been defined as tactile processing with a hedonic or emotional component”.  We agree with the reviewer that affective touch does not necessarily have to mean C-tactile afferent activating touch. We only refer to this type of mean C-tactile afferent touch as an additional argument for the importance of affective touch in social communication. We now clarify this distinction by explicitly providing some examples of affective social touch that do not necessarily involve the activation of C-tactile afferents (such as light touches on the arm or back):  “Affective touch can significantly affect social interactions [9]. For example, a soft touch on the forearm can lead to more favorable evaluations of the toucher [10], a light touch on the back can help to persuade people [11], and caressing touch (e.g., holding hands, hugging, kissing, cuddling, and massaging) can influence our physical and emotional well-being [12]” |
| Relatedly on line 87 – Just because touch is affective it doesn’t have to be social. Is this line referring to discriminative and affective touch, or social and non-social touch, having different neural activations? | We corrected this sentence as follows: “While social and non-social touch events elicit different neural activation patterns” |
| **Methods** – Data Analysis section (lines 187-196): The authors use non-parametric analysis of their ratings data. The approach used to determine assumptions for parametric testing were not met should be reported. | We used a standard ICC analysis, not a non-parametric one. |
| **Discussion** – The discussion is very short and there is scope to go into more depth on the potential utility of this scale for future tactile research. For example, given it combines both valence and arousal, could it be a more sensitive measure for differentiating between evaluations of touch than standard visual analogue scales? This paper provides an example of limitations of a VAS in differentiating between touch on different skin sites - McGlone, F., Olausson, H., Boyle, J. A., Jones‐Gotman, M., Dancer, C., Guest, S., & Essick, G. (2012). Touching and feeling: differences in pleasant touch processing between glabrous and hairy skin in humans. European Journal of Neuroscience, 35(11), 1782-1788. | We thank the reviewer for this interesting suggestion, and we added this suggestion to the discussion:  “A previous study found that slow brush stroking on the forearm and the palm elicited significantly different brain activations, while individual VAS pleasantness and intensity scales were not sufficiently sensitive to distinguish between these two types of touch [69]. Since the EmojiGrid combines both valence and arousal, it would be interesting to investigate whether this instrument is sensitive enough to differentiate between these kinds of touch.” |
| Line 27 -28 – “This affective component of touch plays a significant role in social communication.” – please provide a reference to support this statement. | A reference in support of this statement has been added. |
| Line 33 – suggest “persons” is changed to “people” | corrected |
| Line 83-84 – please provide a reference for the “validated affective image database” referred to here. | We included a reference to the SATED database |
| Line 86-87 – please give term in full on first use before using acronym SATED and provide a reference. | We now provide the full term for the acronym SATED and included a reference |
| Line 221 – p is reported as equal to 0? | P was smaller than 0.0001 |
| Comments of Reviewer 2 | Authors reply |
| 1.1 The introduction addresses general points about affective touch. However, it lacks more detailed literature review on the state of the art studies investigating affective dimension of touch. As an example, the authors introduce in lines 57-70, the SAM test and Likert scales as the state-of-the-art self-assessment tools used in affective studies. I suggest to enlarge this paragraph by discussing the studies using these tools in rating affective touch, the main advantages and disadvantages of these methods and how previous studies tackled the limitations in rating arousal level. | We added some discussion and added several references on the disadvantages that have been observed in the literature and, as far as they have been presented, ways or suggestions to remedy these drawbacks:  “When used to measure the affective appraisal of touch, it appeared that the SAM’s arousal scale is not evident for participants [40]. When participants do not fully un-derstand the meaning of “arousal”, they tend to copy their valence response to the arousal scale (an anchoring effect) [46]. To remedy this effect, participants are some-times provided with additional explanations about this scale, emphasizing that arousal refers to emotional arousal [40]. The use of Likert scales involves cognitive effort, since they require the user to relate experienced emotions to numbers or labels on a scale. As a result, people with stroke related impairments [47] and children [48] are unable to correctly complete VAS scales whereas they can successfully use scales based on iconic facial expressions [49, 50]. The cognitive effort required for the use of VAS scales can also result in an emotion-cognition interaction, such that partici-pants react faster and more consistently to stimuli that are rated as more unpleasant or more arousing than to stimuli that are rated as more pleasant and less arousing [37]. Also, SAM and Likert scales both require two successive assessments of valence and arousal (along two individual scales), making their use more elaborate, error prone and time consuming. Previous studies therefore recognized the need to develop new rating scales for affective touch [51].” |
| The authors justify the use of video clips in eliciting the sense of affective touch to the subjects by evidences in Lines 81 to 94 mentioning " it appears that the brain activity patterns elicited by imagined, perceived and experienced (affective) touch are highly similar [11, 12, 45-47].". I suggest the authors to expand this sentence by discussing more details on the conclusions of these studies. It is not clear and there is no detailed discussion on how the choice of eliciting affective touch by perceiving it could have the similar effect as a real elicitation and what limitations it has. | We added more details on the outcomes of the studies that are mentioned in the text :  “The mere observation of social touch (relative to observation of object touch) acti-vates a number of brain regions (including primary and secondary somatosensory cortices) in a somatotopical way [59], resulting in similar pleasantness ratings [19]. Mental tactile imagery recruits partially overlapping neural substrates in both the primary and secondary somatosensory areas [57]. The anterior insula, which has been implicated in anticipating touch and coding its affective quality [60], responds both to experienced and imagined touch [58]. To some extent, people experience the same touches as the ones they see (they have the ability to imagine how an observed touch would feel [61]): seeing other people’s hands [62], legs [63], neck, or face [59] being touched activated brain regions that also respond when participants are touched on the same body part.” |
| 2) **Results** This section lacks readability, a table reporting the statistical analysis (Median and MAD values and p-values) for a comparative visualisation of the results is missing. | We added a table reporting the median (MAD) valence and arousal ratings, obtained with the SAM and the EmojiGrid, for the (positive, negative and all) social and object touch scenarios from the SATED database. |
| The general outcome of this study suggesting the improvement in rating arousal and valence scales with the proposed self-report tool rather than the standard SAM test is promising for all affective future studies. However, much more investigations in different elicitation protocols should be done to validate the new tool. | We fully agree with the reviewer that many more studies are needed to validate the EmojiGrid. We now include a statement along these lines in the Conclusion section. |
